# Allostimulatory Effects of Dendritic Cells with Characteristic Features of a Regulatory Phenotype

**DOI:** 10.1371/journal.pone.0159986

**Published:** 2016-08-15

**Authors:** M. Kouwenberg, C. W. M. Jacobs, J. van der Vlag, L. B. Hilbrands

**Affiliations:** Department of Nephrology, Radboud university medical center, Nijmegen, the Netherlands; INSERM, FRANCE

## Abstract

**Introduction:**

Tolerogenic dendritic cells (DCs) have the potential to prolong graft survival after transplantation. Tolerogenic DCs are in general characterized by a low expression of co-stimulatory molecule and a high IL-10:IL-12 production ratio. Based on promising results with earlier used alternatively activated DCs, we aimed to generate in culture potentially tolerogenic DC by simultaneously blocking GSK3 by lithium chloride (LiCl) and stimulating TLR2 by PAM_3_CysSerLys_4_.

**Materials and Methods:**

Bone marrow-derived LiClPAM_3_ DCs were generated by the addition of LiCl 24 hours before harvesting, and one hour later PAM_3_CysSerLys_4_. The phenotype of the DCs was assessed by determining the expression of co-stimulatory molecules in flow cytometry and cytokine production in ELISA, whereas their functional properties were tested in a mixed lymphocyte reaction. A fully MHC mismatched heterotopic heart transplant preceded by infusion of donor-derived LiClPAM_3_ DC was performed to assess the tolerogenic potential of LiClPAM_3_ DCs *in vivo*.

**Results:**

LiClPAM_3_ DCs displayed a tolerogenic phenotype accompanied with a low expression of co-stimulatory molecules and a high IL-10:IL-12 production ratio. However, in mixed lymphocyte reaction, LiClPAM_3_ DCs appeared superior in T cell stimulation, and induced Th1 and Th17 differentiation. Moreover, mice pretreated with LiClPAM_3_ DC displayed a reduced graft survival. Analysis of LiClPAM_3_ DC culture supernatant revealed high levels of CXCL-1, which was also found in supernatants of co-cultures of LiClPAM_3_ DC and T cells. Nevertheless, we could not show a role for CXCL-1 in T cell proliferation or activation *in vitro*.

**Discussion:**

LiClPAM_3_ DCs display *in vitro* a tolerogenic phenotype with a high IL-10:IL-12 ratio, but appeared to be highly immunogenic, since allograft rejection was accelerated. As yet unidentified LiClPAM_3_ DC-derived factors, may explain the immunogenic character of LiClPAM_3_ DCs *in vivo*.

## Introduction

Dendritic cells (DCs) are professional antigen presenting cells that can induce activation and differentiation of T cells by a combination of 3 signals: (1) recognition of an antigen-MHC complex by the T cell receptor, (2) engagement of co-stimulatory molecules and, (3) binding of cytokines produced by the DC to cytokine receptors on T cells. While several subsets of DCs are distinguished, including myeloid and plasmacytoid DC, each of these subsets can have either immunogenic or tolerogenic effects. An immunogenic or tolerogenic outcome is mainly determined by the maturation state of the DC, which is reflected by the expression level of co-stimulatory molecules [1]. In numerous animal studies of organ transplantation, prolonged allograft survival has been achieved after injection of tolerogenic, either immature or semi-mature, donor-derived or autologous DCs prior to transplantation [2]. Also in experimental auto-immune diseases, the infusion of tolerogenic DCs has been demonstrated to abate undesirable immune responses [3]. Various strategies and pharmacological agents have been used in DC cultures in order to create tolerogenic DCs. Unifying characteristics of tolerogenic DCs are a low expression of co-stimulatory molecules [4–6], and a favorable cytokine profile in general characterized by a low secretion rate of IL-12 and an increased secretion rate of IL-10. Tolerogenic (human or murine) DCs with an increased IL-10:IL-12 secretion ratio have been generated after culture with different cytokines as IL-10 and TGFβ1 [7,8], and with different pharmacological substances such as glucocorticoids [9,10], vitamin D analogues [11,12], 3-hydroxy-3-methylglutaryl-CoA reductase inhibitors [13], curcumin [14]. In addition, incubation of DCs with several toll like receptor (TLR) ligands such as the TLR2 agonists yeast zymosan [15–17] and Pam_3_CysSerLys_4_ [18], the TLR4 agonist lipopolysaccharide [19], and the TLR5 agonist flagellin [18], has been shown to induce tolerogenic DCs in certain conditions.

In a previous study we observed prolonged allograft survival in a heart transplant model in mice by injecting alternatively activated donor-derived DCs (aaDC, DCs activated with LPS in the presence of dexamethasone) seven days prior to transplantation [20]. Indeed, these aaDC were characterized by a low expression of the co-stimulatory molecules CD40 and CD86, and an increased IL-10:IL-12 secretion ratio. IL-10 is an anti-inflammatory cytokine, especially produced by DCs upon TLR2 triggering and regulatory T and B cells. The production of IL-10 after TLR2 ligation results from the activation of phosphatidylinositol 3-OH kinase (PI3K) and subsequent inhibition of glycogen synthase kinase 3 (GSK3). Pharmacologic inhibition of GSK3 together in combination with TLR2 triggering results in enhanced IL-10 production and abrogated IL-12 production [21]. The cytokine profile and consequently the tolerogenic potential of DCs can thus be manipulated by specific TLR triggering and simultaneously targeting intracellular signal transduction pathways. These findings led us to hypothesize that bone marrow-derived mouse DCs, cultured in the presence of a TLR2-agonist (PAM_3_CysSerLys_4_) and a GSK3 inhibitor (lithium chloride, LiCl), could have strong tolerogenic properties rendering them suitable for extensive prolongation of graft survival in a transplant model. We were indeed able to culture DCs with a highly tolerogenic phenotype, characterized by a low expression of co-stimulatory molecules and a high IL-10:IL-12 production ratio. Despite these favorable characteristics, however, the LiClPAM_3_ DCs turned out to superiorly stimulate T cell proliferation in mixed lymphocyte reaction and to cause accelerated graft rejection in a (heart) transplant model.

## Materials and Methods

### Mice

Experiments were performed with completely MHC-mismatched combinations of male C57Bl/6N (donor, H-2^d^) and male Balb/c (graft recipient, H-2^b^) mice. All mice were obtained from Charles River Laboratories (USA), aged 6 to 8 weeks old. All animal experiments were carried out after permission granted by the animal ethics committee of the Radboud University Nijmegen (Permit Number 2011–024). Animals were housed under specified pathogen-free conditions and handled according to the guidelines of the local animal welfare body of the Radboud University Nijmegen.

### Cell culture

#### Dendritic cell culture

Donor dendritic cells were cultured from bone marrow derived cells according to a protocol adapted from Lutz et al. [22,23]. Briefly, C57Bl/6N mice were euthanized by cervical dislocation, femora and tibia were harvested and bone marrow was flushed with medium consisting of RPMI-1640 Dutch modification (Invitrogen, Carlsbad, USA) supplemented with 50 μM β-mercaptoethanol (Sigma-Aldrich, St Louise, USA), 1% glutamax, 1% pyruvate and 10.000 U/ml penicillin-streptomycin (all Invitrogen).

Cells were suspended and subsequently cultured in six well plates (0.8 x 10^6^ cells/well; Corning Incorporated, USA) containing medium supplemented with 10% fetal calf serum (FCS, BioWhittaker, Lonza, Walkersville, USA) and 20 ng/ml rGM-CSF (PeproTech, Rocky Hill, USA). Cells were incubated at 37°C and 5% CO_2_ for 9 days. To obtain LiClPAM_3_ DCs, 10 mM LiCl (L-7026, Sigma-Aldrich Chemie, Steinheim, Germany) was added on the 8^th^ day of culture and one hour later 1 μg/ml PAM_3_CysSerLys_4_ (PAM_3_, tlrl-pms, InvivoGen, San Diego, USA) was added. Mature DCs were generated by adding 1 μg/ml ultra pure lipopolysaccharide (ultra pure LPS from E. coli 0111:B4, Invivogen, San Diego, USA) to untreated cells 24 hours before harvesting. After 9 days of culture, DC were harvested. Culture supernatant was stored at -20°C for cytokine measurement later on, cells were used for flow cytometric analysis, mixed lymphocyte reaction or for intravenous administration to Balb/c mice prior to transplantation.

#### Mixed lymphocyte reaction

In mixed lymphocyte reactions (MLR), a protocol adapted from earlier performed MLR [24] was used and 2x10^5^ responder cells (Balb/c) were co-cultured with 5x10^4^ stimulator cells (C57Bl/6N). To prepare responder cells the spleen was pushed through a sterile stainless wire mesh (70 μmeter) and erythrocytes were lysed by Ammonium-Chloride-Potassium lysing buffer. Afterwards, a negative selection was performed with anti-MHC class II microbeads and a LS column (Miltenyi Biotec GmbH, Gladbach, Germany). Before selection, the splenocytes contained 38% T cells and 50% B cells. After selection, the negative fraction consisted of 82% T cells and 5% B cells. Splenocytes or enriched T cells were intracellular labeled with CFSE (Molecular Probes, Life Technologies Ltd, Paisley, UK) according to the manufacturer’s instructions. Stimulator cells consisted of dendritic cells, cultured as described above and washed three times before co-culture with the responder cells. Responder and stimulator cells were co-cultured in medium supplemented with 10% FCS as described above in a 96-wells round bottom plate (Costar, Corning Inc. USA). Where indicated, αCXCL-1 (Clone 48415, R&D Systems) or isotype control (Rat IgG2_A_, R&D Systems), or a selective CXCR2-antagonist, SB225002 (Cayman Chemical, Michigan USA) or vehicle (sterile DMSO, Sigma Aldrich) was added to the MLR.

Cells were incubated at 37°C, 95% humidity and 5% CO_2_. At day 4 and 6 of culture, cells were harvested. Culture supernatant was collected and stored at -20°C for cytokine analysis. Proliferation of responder cells was measured by dilution of CFSE signal by flow cytometry.

### Heart transplantation

Seven days prior to heart transplantation, graft recipients (Balb/c mice) were intravenously (tail vein) infused with 10^6^ C57Bl/6N DCs of one of the three different types (control, LPS or LiClPAM_3_). Before surgery, C57Bl/6N donor mice were anesthetized by subcutaneous injection of FFM-mix (fentanyl citrate, fluanisone, midazolam, 0.2ml per 10 grams of body weight). Recipient mice (Balb/c) were anesthetized by isoflurane inhalation and subcutaneous injection of FFM-mix (0.1 ml per 10 grams of body weight). The heart graft was placed in a heterotopic position (intra-abdominal) according to the technique described by Corry et al. [25]. After surgery, intramuscular injection of buprenorfine was used as analgesic medication. Graft survival was monitored by daily transabdominal palpation of the donor heart. In case of strong decrease in strength of heart pulsation or absent pulsation, rejection was scored. Mice were daily monitored for transplant function, weight and well being (scored for general appearance and behavior, appearance of fur and mucous membranes, respiration rate and signs of dehydration). After a follow up of 18 days, mice were euthanized by cervical dislocation. Ten percent of the included mice did not reach the experimental endpoint because they died suddenly or were euthanized after reaching a human endpoint.

### Antibodies

In flow cytometry (BD FACS Calibur, New Jersey, USA) the following monoclonal antibodies were used for cell staining: CD3 (clone 17A2, rat monoclonal, diluted 1:100, BD Biosciences, New Jersey, USA), CD11c (clone N418, hamster monoclonal, diluted 1:100, ABD Serotec, Kidlington, UK), CD19 (clone 1D3, rat monoclonal, diluted 1:100, BD Biosciences), CD40 (clone FGK45.5, rat monoclonal, diluted 1:40, Miltenyi), CD86 (clone PO3.1, rat monoclonal, diluted 1:80, eBioscience), CD80 (clone 16-10A1, rat monoclonal, diluted 1:400, Biolegend, Fell, Germany), MHCII (clone M5/114.15.2, rat monoclonal, diluted 1:800, eBioscience). All used antibodies have been validated by others [22]. The cytokines IL-1β, IL-4, IL-6, IL-10, IL-12, IL-17, IL-21, IL-23, TNF-α, IFN-γ (all eBioscience) and CXCL-1 (R&D systems, Minneapolis, USA) were measured in supernatants of bone marrow derived DC culture and MLR culture by using ELISA kits according to the manufacturer’s instructions.

A protein-based mouse cytokine/chemokine antibody array (Ray Biotech Inc, Norcross, USA) was performed on DC culture supernatant for comparative analysis of levels of a broad panel of 120 chemokine, cytokine and growthfactors in pooled supernatants of different types of DC cultures.

### Statistical analysis

For statistical analysis GraphPad Prism (version 5.0 for Windows, GraphPad Software, San Diego, USA) was used. Results are expressed as mean ± SD. For comparison between two groups the Mann-Whitney U test was used. Graft survival was compared using Kaplan-Meier curves and the log rank test. A P-value < 0.05 was considered significant.

## Results

### Combined TLR2 triggering and GSK3 inhibition generates DCs with features of a tolerogenic phenotype

**Fig 1:**
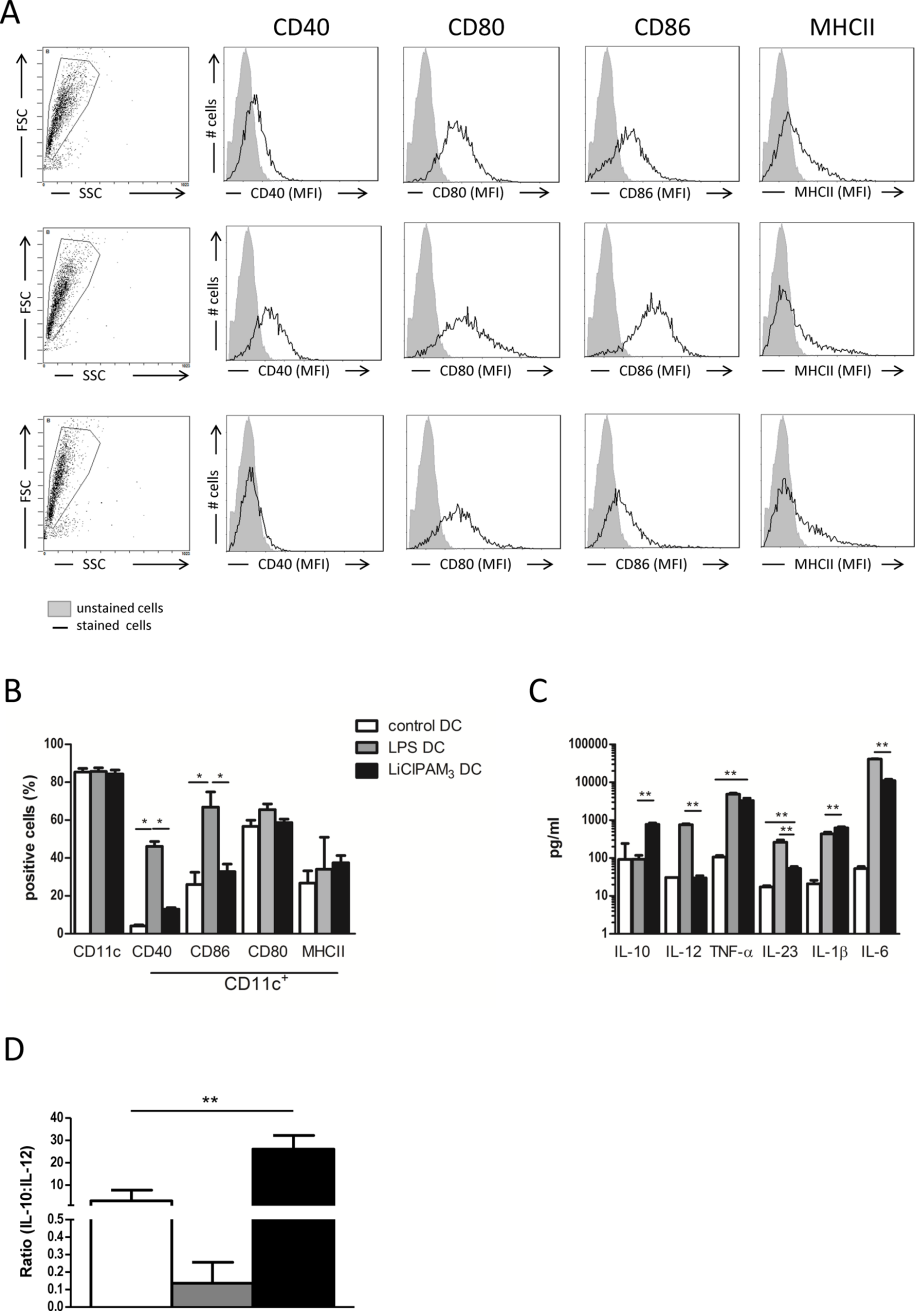
LiClPAM_3_ dendritic cells (DCs) display characteristic features of a tolerogenic phenotype with high IL-10:IL-12 ratio. Bone marrow-derived C57Bl/6N DCs were cultured for 9 days with GM-CSF. Cells were gated for CD11c+ (A) and subsequently analyzed by flow cytometry for percentage of CD40, CD80, CD86, and MHCII cells (black lines, grey area depicts unstained cells). The percentage of CD40+ or CD86+ cells was lower in LiClPAM_3_ DC compared to LPS DC (* p<0.05) (B). Supernatants obtained after 9 days of DC culture were analyzed for cytokines (** p<0.01) (C). LiClPAM_3_ DCs have a higher IL-10:IL-12 ratio compared to LPS and control DC (** p<0.01) (D). Results are of 6 independent experiments.

Immature bone marrow derived DC were treated with LiCl to inhibit GSK3 and subsequently stimulated with the TLR2 ligand PAM_3_CysSerLys_4_ (LiClPAM_3_ DC). Like untreated immature DCs, a low percentage of the LiClPAM_3_ DCs was positive for the co-stimulatory molecules CD40 and CD86 (Fig 1A and 1B). After stimulation with LPS, an expected strong increase in the percentage of CD40+ and CD86+ cells was observed. The expression of CD80 and MHCII was comparable between LiClPAM_3_ DCs and untreated immature DCs.

The cytokine secretion by the various types of DCs was measured in the supernatants obtained at day 9 of DC culture (Fig 1C). As compared to immature control DC, LiClPAM_3_ DCs produced much higher levels of IL-10, while levels of IL-12 were equally low, resulting in a high IL-10:IL-12 ratio (Fig 1D) fitting with a tolerogenic potential. An opposite profile with high IL-12 levels and low IL-10 levels was observed after stimulation with LPS. While IL-1β levels in LiClPAM_3_ DC culture supernatant exceeded that of LPS DC, levels of IL-6 and IL-23 were significantly lower. TNF-α levels were comparable in the supernatants of LPS and LiClPAM_3_ DCs, while levels of IL-4, IL-21 and interferon-γ were undetectable. Although DCs treated with LiCl or PAM_3_ alone also displayed a low expression of CD40 and CD86, their IL-10:IL-12 ratio was lower than that of the LiClPAM_3_ DC (S1 Fig).

Taken together, the cultured LiClPAM_3_ DCs display characteristic features of a tolerogenic phenotype with a low expression of co-stimulatory molecules and a high IL-10:IL-12 ratio.

### LiClPAM_3_ DCs are potent T cell stimulators and induce T_H_1 and T_H_17 differentiation

In an allogeneic mixed lymphocyte reaction, the stimulatory capacity of the LiClPAM_3_ DC was analyzed. MHCII-depleted, CFSE stained Balb/c splenocytes were co-cultured with C57Bl/6N DCs for 3–6 days.

**Fig 2:**
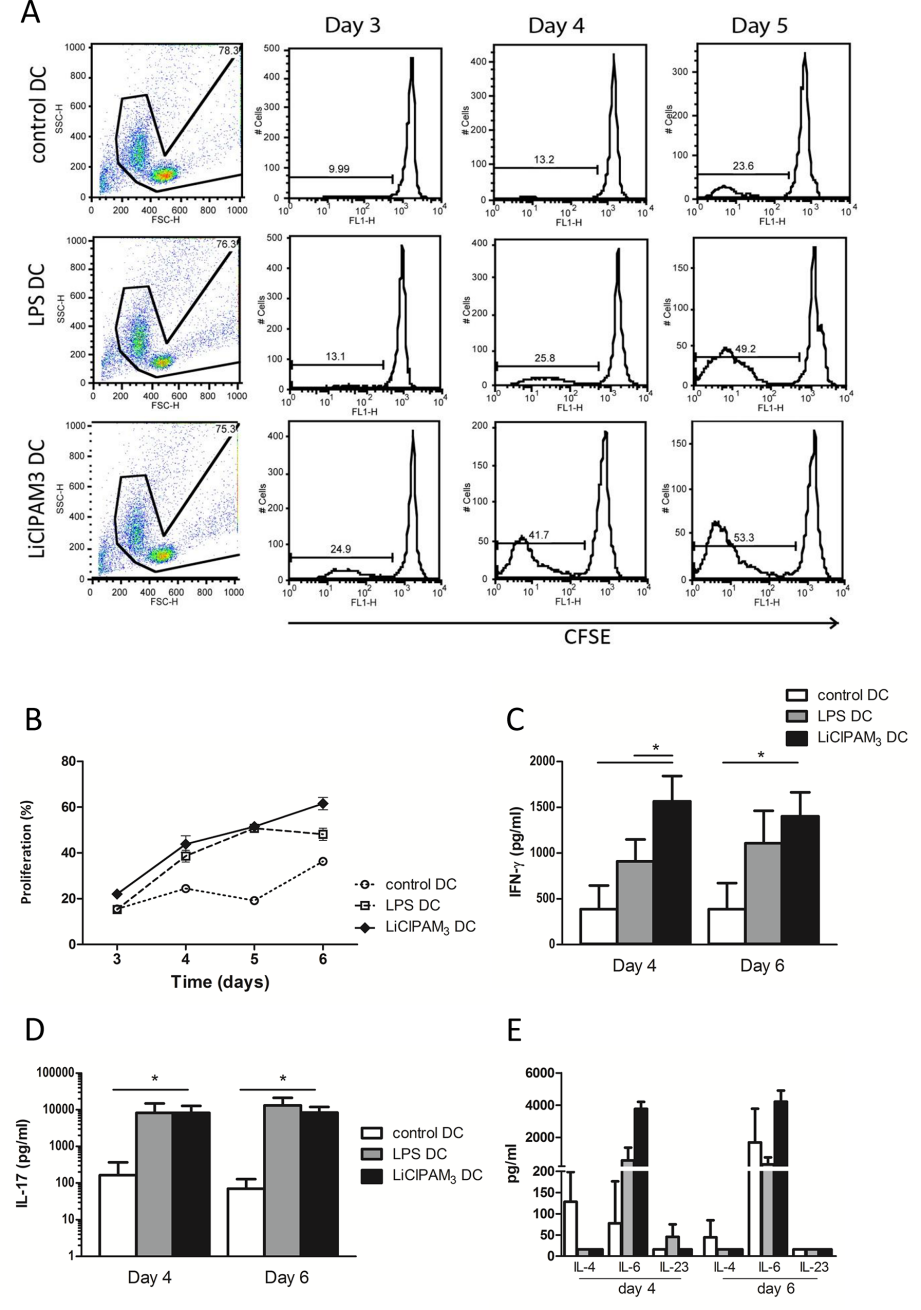
LiClPAM_3_ dendritic cells (DCs) are potent T cell stimulators and induce Th1 and Th17 differentiation *in vitro*. The T cell stimulatory capacity of (C57Bl/6N) LiClPAM_3_ DCs was tested in mixed lymphocyte reaction. (Balb/c) T cell proliferation was analyzed by dilution of CFSE signal (A). LiClPAM_3_ DC stimulation resulted in higher T cell proliferation at days 3, 4, 5, and 6 compared to control DC stimulation (*p<0.05) (B). T cell differentiation was analyzed by measuring cytokine in co-culture supernatants in ELISA. Interferon-γ (C), but not IL-17 (D) production, by LiClPAM_3_ DC stimulated T cells was higher compared to LPS DC stimulated T cells at day 4 (* p<0.05). IL-4, IL-6 and IL-23 levels did not differ between groups (E). Results are of 3 independent experiments.

As expected, stimulation with immature DCs resulted in low proliferative T cell responses (Fig 2A). Surprisingly, in cultures with LiClPAM_3_ DCs we observed high proliferative T cell responses, which were similar to that after stimulation with LPS DCs (Fig 2B). Upon LiClPAM_3_ DC stimulation, T cells produced mainly IFN-γ (Fig 2C) and IL-17 (Fig 2D), suggesting a shift towards Th1 and Th17 differentiation. Comparable levels of IFN-γ and IL-17 were found in supernatants of co-cultures containing LPS DCs, whereas levels of IL-10, IL-21 and TNF-α were undetectable.

Thus, *in vitro*, LiClPAM_3_ DCs display an immunogenic character, demonstrated by strong T cell stimulatory capacity and interferon-γ and IL-17 production in co-culture.

### Pretreatment of recipients with LiClPAM_3_ DCs results in accelerated heart graft rejection

**Fig 3:**
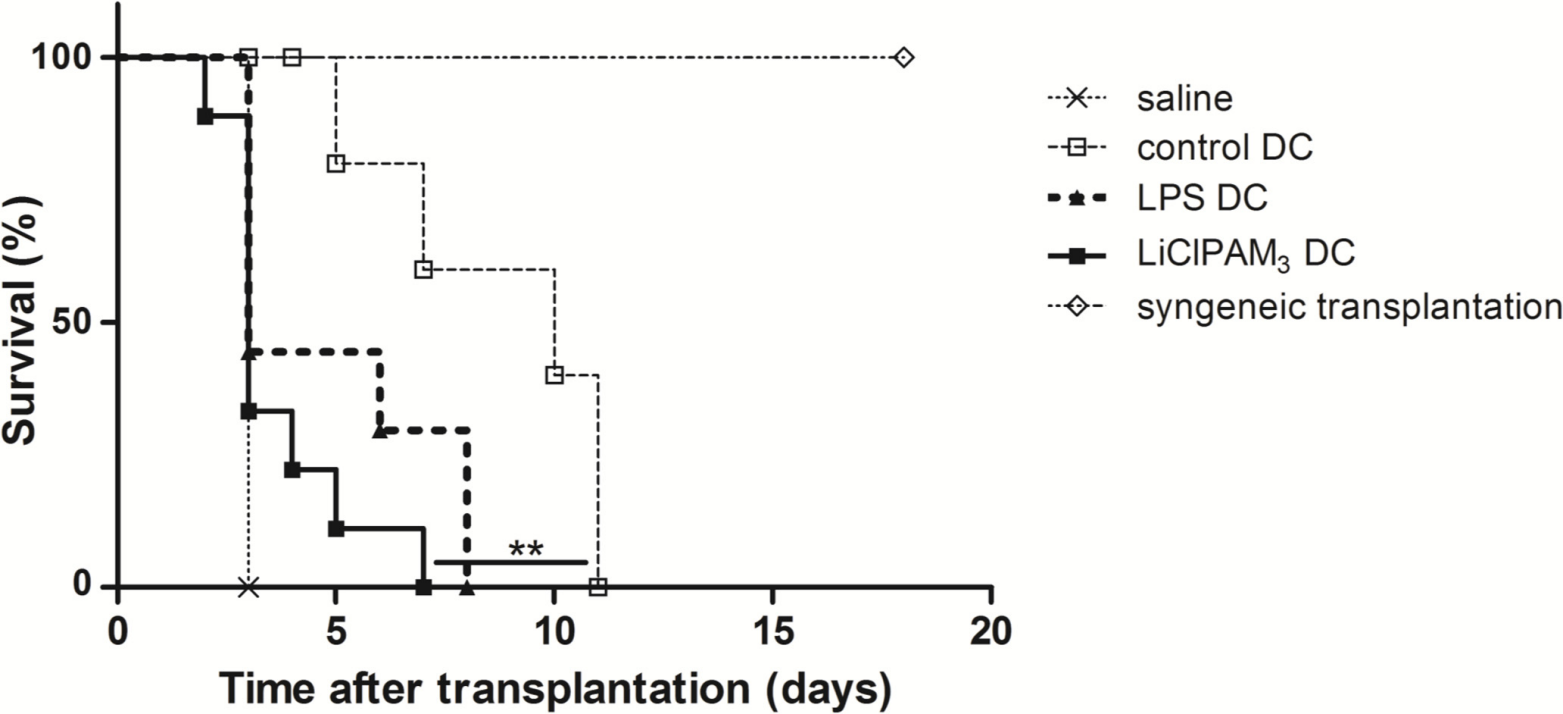
LiClPAM_3_ DC pre-treatment results in accelerated allograft rejection in a fully MHC mismatched heterotopic heart transplant model. Balb/c recipient mice were intravenously infused with 10^6^ C57Bl/6N donor-derived DC or syngeneic DC seven days prior to transplantation. Graft survival was daily assessed by transabdominal palpation of the donor heart. LiClPAM_3_ DC pre-treatment (n = 9) resulted in accelerated allograft rejection compared to control DC pretreated mice (n = 10) (**p<0.01).

To test the effects of the phenotypic tolerogenic LiClPAM_3_ DCs *in vivo*, we pretreated mice with donor-derived DCs and performed a fully MHC-mismatched heterotopic vascularized heart transplantation. Pretreatment of recipients with LiClPAM_3_ DCs resulted in a median graft survival of 3 days (n = 9), which was comparable to graft survival after pretreatment with mature LPS DCs (median graft survival of 4 days, n = 9), though significantly shorter than after pretreatment with immature control DC (median graft survival of 10 days (n = 10; Fig 3). Syngeneic transplanted mice, pretreated with LiClPAM_3_ DCs (n = 4), survived until the end of follow up.

In conclusion, the LiClPAM_3_ DCs appeared to be strongly immunogenic *in vivo* with accelerated graft rejection.

### LiClPAM_3_ DCs produce high amounts of the chemokine CXCL-1

**Fig 4:**
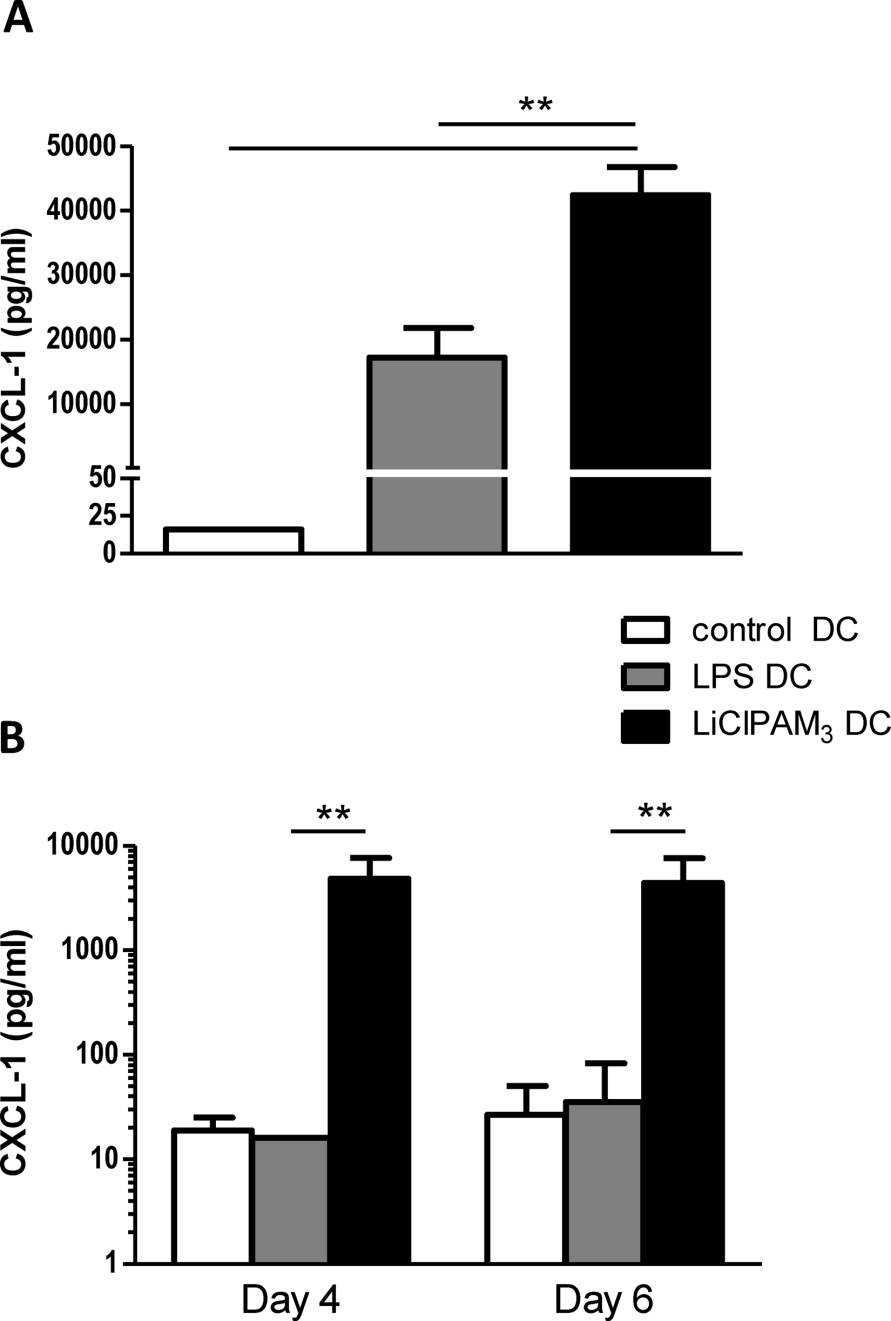
LiClPAM_3_ DC produce high levels of CXCL-1 in culture, which sustains in co-culture with MHCII-depleted splenocytes. CXCL-1 was measured in (C57Bl/6N) DC culture supernatant after 9 days of culture (A). LiClPAM_3_ DC produce large amounts of CXCL-1 during culture, significantly higher as compared to LPS DC (**p<0.01). In supernatant of LiClPAM_3_ DC stimulated (Balb/c) T cells (B) higher levels of CXCL-1 were found as compared to LPS DC stimulated conditions (** p<0.01). Results are of 5 independent experiments.

While several features of the LiClPAM_3_ DCs suggested a tolerogenic potential, *in vitro* and *in vivo* experiments revealed robust allostimulatory capacity. To unravel this unexpected discrepancy, further analysis of cytokine, chemokine and growth factor production by LiClPAM_3_ DCs was performed using a protein-based cytokine/chemokine array, followed by ELISA of cytokines and chemokines of interest. The cytokine/chemokine array indicated relatively high levels of CXCL-1, IL-12, CCL-11, CXCL-5, CXCL-11 and soluble TNF-α receptor type 1 (sTNF-α RI) in the culture supernatant of LiClPAM_3_ DCs (S1 Table). Based on literature data on the role of these proteins in DC-T cell interaction and Th1/TH17 differentiation [26–28], we focused on CXCL-1 and confirmed with ELISA high levels of CXCL-1 in LiClPAM_3_ DC culture supernatant, significantly higher than in supernatants of LPS or control DC (Fig 4A). We next-analyzed MLR supernatants and again found high levels of CXCL-1 in LiClPAM_3_ DC stimulated culture supernatants, whereas CXCL-1 levels appeared undetectable in culture supernatants of LPS DC and control DC stimulated conditions (Fig 4B).

### LiClPAM_3_ DC-derived CXCL-1 is not responsible for the proliferative T cell response

Based on the immunogenic character of the LiClPAM_3_ DC combined with high production of CXCL-1, we postulated that LiClPAM_3_ DC-derived CXCL-1 could induce T cell proliferation.

To test our hypothesis, we performed MLRs in which we neutralized LiClPAM_3_ DC-derived CXCL-1 with anti-CXCL-1 or blocked CXCR2, the receptor of CXCL-1.

**Fig 5:**
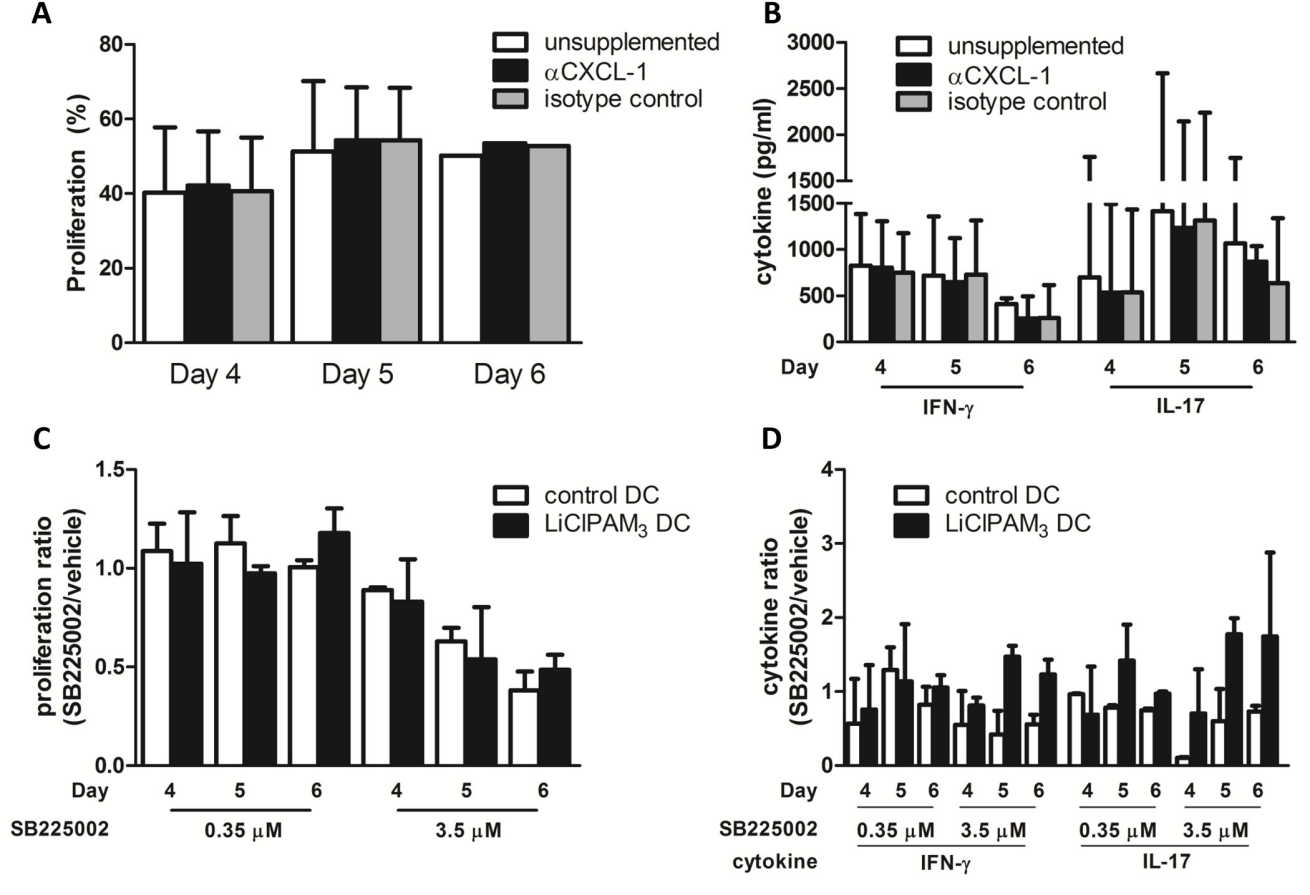
LiClPAM_3_ DC derived CXCL-1 is not involved in T cell activation or proliferation. Balb/c MHCII-depleted splenocytes were co-cultured for 4–6 days with C57Bl/6N LiClPAM_3_ DC in the presence of a CXCL-1 neutralizing antibody, or an isotype control. Neutralization of DC derived CXCL-1 did not affect T cell proliferation (A) or activation (B) (n = 5). Selective chemical CXCR2 blockade by SB225002 did not result in reduced T cell proliferation (C) or activation (D) caused by CXCR2 blockade, compared to vehicle only (n = 3).

First, we neutralized DC derived CXCL-1 by adding anti-CXCL-1 (or isotype control) to co-cultures of LiClPAM_3_ DC and MHCII-depleted splenocytes. Neutralization of DC derived CXCL-1 did not influence T cell proliferation (Fig 5A) or activation (Fig 5B). In addition, selective blockade of CXCR2 by the chemical substance SB225002 could not abolish the proliferative response (Fig 5C) or activation (Fig 5D) of T cells either.

In conclusion, although the immunogenic LiClPAM_3_ DC were characterized by the production of high levels of CXCL-1, we were not able to show that LiClPAM_3_ DC-derived CXCL-1 is directly responsible for the increased T cell activation and proliferation.

## Discussion

Cellular vaccination with tolerogenic DCs prior to transplantation to create donor-specific tolerance is a promising approach in transplantation medicine [2]. Characteristic features of these tolerogenic DCs are a high IL-10:IL-12 secretion ratio and low expression of co-stimulatory molecules, which drives T cell differentiation towards regulatory T cells and T cell anergy.

Previously, we generated alternatively activated DCs by adding dexamethasone and LPS, which resulted in DC with a low CD40, CD86 and MHCII expression and an increased IL-10:IL-12 ratio compared to both immature and LPS-matured DCs [20]. In tolerogenic DCs described in literature a decreased secretion of other pro-inflammatory cytokines, such as TNF-α, IL-6, and an increased secretion of other anti-inflammatory cytokines, such as TGF-β, has been reported as well. Nevertheless, the majority of studies mention a low expression of co-stimulatory molecules and a high IL-10:IL-12 ratio.

Based on the finding that combined TLR2 triggering and GSK3 inhibition results in a high IL-10:IL-12 production ratio [21], we manipulated bone marrow-derived dendritic cells in culture by adding LiCl (inhibitor of GSK3) and PAM_3_CysSerLys_4_ (TLR2 agonist). Indeed, a low percentage of the generated LiClPAM_3_ DCs expressed co-stimulatory molecules and the cells displayed a high IL-10:IL-12 secretion ratio. Despite these characteristic features of a tolerogenic phenotype, LiClPAM_3_ DCs appeared to have a highly immunogenic character *in vitro*. When these DCs were used in co-cultures with responder lymphocytes, high levels of IL-17 and interferon-γ were found. Based on the proliferative response of the lymphocytes, we interpret this as a skewing towards Th1 and Th17 subsets, although both IL-17 and interferon-γ producing (Foxp3+) regulatory T cell populations have been demonstrated in humans [26–28]. Since IL-12 production by LiClPAM_3_ DC is low, we suggest that the increased production of IL-1β may have contributed to this Th17 shift, since this cytokine has the potential to direct naive T cells towards Th17 [29,30]. The *in vitro* immunogenic character of the LiClPAM_3_ DC was confirmed *in vivo* by accelerated graft rejection after LiClPAM_3_ DC infusion prior to heart transplantation. Since we found high levels of the chemokine CXCL-1 in both LiClPAM_3_ DC cultures and supernatants of MLRs where these DCs were used as stimulators, we speculated that CXCL-1 was responsible for the increased T cell response and accelerated allograft rejection. CXCL-1, also called KC or GRO-α, is mainly known as an important regulator of neutrophil recruitment [31] and activation [32]. The sole receptor for CXCL-1 is CXCR2, which is reported to be expressed by mouse T cells [33,34]. However, further analysis could not substantiate a direct role for CXCL-1 in T cell activation or proliferation. Likely, another LiClPAM_3_ DC derived factor, soluble or cell bound, is responsible for the unexpected immunogenic character of the LiClPAM_3_ DC.

Glycogen synthase kinase 3 is a ubiquitous serine/threosine kinase involved in multiple cellular functions, as cell signaling [35], cell division [36] and cell proliferation [37]. By regulation of the transcription factors nuclear factor κB (NFκB) [38], cAMP response element-binding protein (CREB), and activator protein-1 (AP-1) [39], the isoform GSK3β directly influences expression of pro- and anti-inflammatory cytokines [21]. Therefore, GSK3β regulates both innate and adaptive immunity [40]. Focusing on DC and T cell functioning, GSK3 inhibition has recently been associated with increased IL-10 production in different T helper cell subsets [41] and reduced TNF-α production by human lymphocytes [42]. GSK3 inhibition in dendritic cells has been associated with both interferon-β and LPS induced IL-10 production [43,44], while IL-12p40 production was decreased in GSK3β1 silenced (but not LiCl treated) and TLR2 triggered (bovine) endothelial cells [45]. Beneficial anti-inflammatory effects of GSK3 inhibition have been observed in several animal models, like LPS and *Klebsiella pneumonia-* induced sepsis [21,46], LPS-induced acute renal failure [47], and traumatic tissue injury [48,49]. Based on these data, TLR2 triggering combined with GSK3 inhibition has in theory the potential to induce tolerogenic DC. While the phenotype of the cultured LiClPAM_3_ DCs we generated supported this hypothesis, this was not confirmed by the functional characteristics of these cells, which stresses the importance of performing adequate functional (*in vivo*) tests when evaluating the immunomodulatory potential of various cell types.

Collectively, although TLR2 triggering and GSK3 inhibition increased the IL-10:IL-12 secretion ratio, this did not result in a desired tolerogenic capacity of LiClPAM_3_ DCs. Our results indicate that the LiClPAM_3_ DCs have a direct influence on T cell function and differentiation, though not mediated by CXCL-1. In an attempt to generate donor-specific tolerance we focused on the co-stimulatory molecule expression and cytokine profile of DC, since these factors are known to direct T cell differentiation during DC–T cell interaction [50]. Although DC derived IL-10 is capable in skewing a naïve T cell towards a regulatory phenotype [51], other cytokines may override the tolerogenic effect of IL-10. LiClPAM_3_ DCs produce besides IL-10 also pro-inflammatory cytokines as IL-1β, IL-6, TNF-α and IL-12. It is therefore most likely that the composition of the resulting cytokine and chemokine cocktail has determined the immunogenic character of the LiClPAM_3_ DCs in MLR *in vitro* and experimental transplantation *in vivo*.

## Supporting Information

S1 Fig IL-10:IL-12 ratio in supernatant of cultured control, lithium chloride (LiCl), PAM_3_ or LiCl PAM_3_ treated (C57Bl/6N) bone marrow derived dendritic cells after 9 days of culture. (TIF)

S1 Table Protein array results of dendritic cell culture supernatant. (DOCX)
