# Supplementary figures and images for: Allostimulatory Effects of Dendritic Cells with Characteristic Features of a Regulatory Phenotype
Source: PLoS One. 2016 Aug 15;11(8):e0159986. doi: 10.1371/journal.pone.0159986 (PMC4985155; doi:10.1371/journal.pone.0159986)

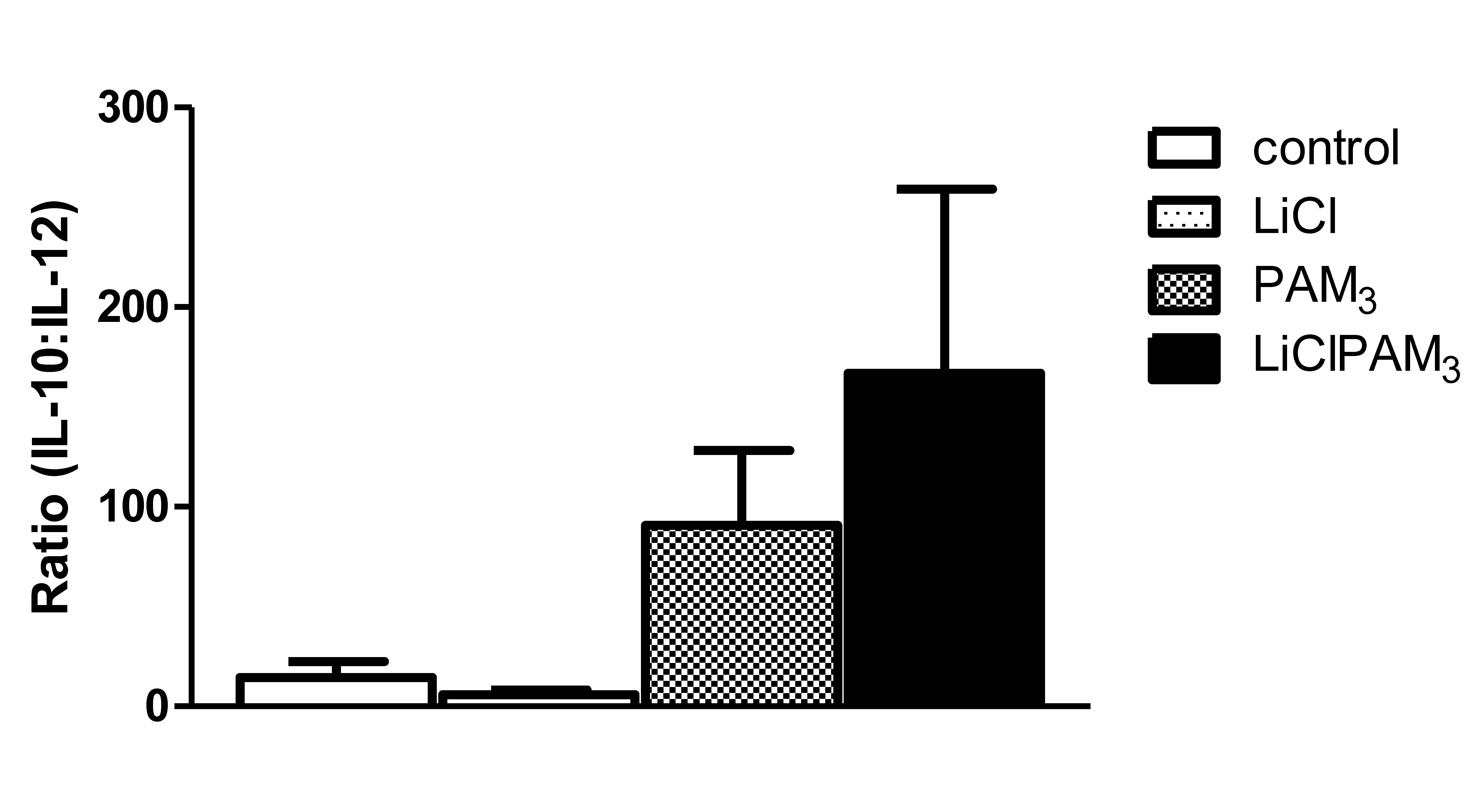

Supplement: S1 Fig — (TIF) [file pone.0159986.s001.tif]
