# Supplementary material for: Allostimulatory Effects of Dendritic Cells with Characteristic Features of a Regulatory Phenotype
Source: PLoS One. 2016 Aug 15;11(8):e0159986. doi: 10.1371/journal.pone.0159986 (PMC4985155; doi:10.1371/journal.pone.0159986)
Supplement: S1 Table — (DOCX) [file pone.0159986.s002.docx]

S1 Table: Protein array results of dendritic cell culture supernatant.

| Protein | Synonym | Dendritic cell culture supernatant | | | Ratio (signal intensity) | |
| --- | --- | --- | --- | --- | --- | --- |
|  |  | Control | LPS | LiClPAM_3_ | LiClPAM_3_/control | LiClPAM_3_/LPS |
| IL-1α |  | ND | 7.27 | 6.78 | - | 0.93 |
| IL-1β |  | ND | 5.89 | ND | - | - |
| IL-2 |  | 5.44 | ND | 5.23 | 0.96 | - |
| IL-3 |  | ND | ND | 6.63 | - | - |
| IL-4 |  | 7.28 | ND | 8.48 | 1.16 | - |
| IL-5 |  | ND | 8.74 | 6.44 | - | 0.74 |
| IL-6 |  | ND | 34 | 25.7 | - | 0.76 |
| IL-9 |  | 6.48 | 9 | 6.39 | 0.99 | 0.71 |
| IL-10 |  | ND | ND | ND | - | - |
| IL-12p40/p70 |  | ND | 27.7 | 23.3 | - | 0.84 |
| IL-12p70 |  | 6.78 | 10 | 6 | 1.13 | 1.67 |
| IL-13 |  | 7.07 | ND | ND | - | - |
| TNF-α |  | ND | 5 | ND | - | - |
| CCL-1 |  | 6 | 11.3 | 7.83 | 1.24 | 0.69 |
| CCL-2 |  | 6.88 | 8.48 | 9.57 | 1.39 | 1.13 |
| CCL-3 |  | 9.61 | 8.05 | 8.31 | 0.86 | 1.03 |
| CCL-5 |  | 7 | 14 | 9 | 1.39 | 0.64 |
| CCL-9 |  | 29.8 | 26.3 | 29.6 | 0.99 | 1.13 |
| **CCL-11** |  | **6.54** | **5** | **7** | **1.00** | **1.22** |
| CCL-12 |  | 7.39 | 8.24 | 7.14 | 0.97 | 0.87 |
| CCL-17 |  | 13.5 | 13.8 | 15.4 | 1.14 | 1.12 |
| CCL-19 |  | 9.96 | 10.2 | 9.25 | 1.02 | 1.10 |
| CCL-20 |  | 8.22 | 8.72 | 8.32 | 1.01 | 0.95 |
| CCL-24 |  | 5.03 | 8.58 | ND | - | - |
| CCL-25 |  | ND | 6.49 | 6.07 | - | 0.94 |
| CCL-27 |  | 7.57 | 9.67 | 10.7 | 1.41 | 1.11 |
| **CXCL-1** |  | **ND** | **16** | **28** | **-** | **1.44** |
| CXCL-2 |  | 11.2 | 27.7 | 29.5 | 2.63 | 1.06 |
| CXCL-4 |  | 10.6 | 8.72 | 8.22 | 0.78 | 0.94 |
| **CXCL-5** |  | **7** | **8.63** | **13** | **1.75** | **1.51** |
| CXCL-9 |  | ND | 5.59 | ND | - | - |
| CXCL-10 |  | ND | 6.67 | ND | - | - |
| **CXCL-11** |  | **6.6** | **5** | **7** | **1.01** | **1.22** |
| CXCL-12 | SDF-1 | ND | 6 | ND | - | - |
|  | SDF-1α | 7.08 | ND | 6.51 | 0.92 | - |
| CXCL-13 |  | 6 | 9 | 5.97 | 1.08 | 0.70 |
| CXCL-16 |  | ND | 6.49 | 6.07 | - | 0.94 |
| XCL1 |  | 7.31 | 9 | 9 | 1.27 | 1.05 |
| CX3CL1 |  | 5.43 | 9 | 5 | 1.00 | 0.64 |
| CD30L |  | 7 | 8.59 | 7 | 0.99 | 0.76 |
| GSCF |  | 6.4 | 6.53 | 6.59 | 1.03 | 1.01 |
| GM-CSF |  | 7 | 5.95 | 6 | 0.82 | 0.98 |
| IGFBP-3 |  | 8.08 | 6.93 | ND | - | - |
| Leptin |  | 5.46 | 5 | 6 | 1.12 | 1.15 |
| Leptin R |  | 6.84 | ND | 6.58 | - | - |
| MCSF |  | 7 | 9 | 9.65 | 1.41 | 1.12 |
| P-selectin |  | 10.2 | 8.83 | 8.13 | 0.80 | 0.92 |
| TIMP-1 |  | 9 | 8.63 | 9 | 1.01 | 1.04 |
| **sTNF-α RI** |  | **7.69** | **6.5** | **10** | **1.24** | **1.47** |
| sTNF-α RII |  | 6 | 10 | 9 | 1.48 | 0.95 |
| TPO |  | 6.92 | ND | 7.45 | 1.08 | - |
| VCAM-1 |  | 7.16 | ND | ND | - | - |
| VEGF |  | 6.91 | 6.46 | 6.9 | 1.00 | 1.07 |

ND, not detectable

Table shows the signal intensity (per dendritic cell condition) of all cytokines, chemokines and growthfactors that could be detected in the performed protein array. Ratio’s shown reflect signal intensity ratio of LiClPAM_3_ versus control DC, and LiClPAM_3_ versus LPS DC. Bold factors were – based on the increased ratio’s - considered as interesting
